# Whites but Not Blacks Gain Life Expectancy from Social Contacts

**DOI:** 10.3390/bs7040068

**Published:** 2017-10-16

**Authors:** Shervin Assari

**Affiliations:** 1 Center for Research on Ethnicity, Culture and Health (CRECH), School of Public Health, University of Michigan, Ann Arbor, MI 48109-2029, USA; assari@umich.edu; 2 Department of Psychiatry, University of Michigan, Ann Arbor, MI 48109, USA

**Keywords:** racial health disparities, social isolation, social engagement, mortality

## Abstract

*Background*. Recent research suggests that the health gain from economic resources and psychological assets may be systematically larger for Whites than Blacks. *Aim*. This study aimed to assess whether the life expectancy gain associated with social contacts over a long follow up differs for Blacks and Whites. *Methods*. Data came from the Americans’ Changing Lives (ACL) Study, 1986–2011. The sample was a nationally representative sample of American adults 25 and older, who were followed for up to 25 years (*n* = 3361). Outcome was all-cause mortality. The main predictor was social contacts defined as number of regular visits with friends, relatives, and neighbors. Baseline demographics (age and gender), socioeconomic status (education, income, and employment), health behaviors (smoking and drinking), and health (chronic medical conditions, obesity, and depressive symptoms) were controlled. Race was the focal moderator. Cox proportional hazard models were used in the pooled sample and based on race. *Results*. More social contacts predicted higher life expectancy in the pooled sample. A significant interaction was found between race and social contacts, suggesting that the protective effect of more social contacts is smaller for Blacks than Whites. In stratified models, more social contacts predicted an increased life expectancy for Whites but not Blacks. *Conclusion*. Social contacts increase life expectancy for White but not Black Americans. This study introduces social contacts as another social resource that differentially affects health of Whites and Blacks.

## 1. Background

According to *Blacks’ diminished return* [1,2], minority status may bound the potential health gains from available psychosocial resources [3,4,5,6,7,8]. As a result, Whites gain more health from several resources and assets such as education [9], employment [10], and neighborhood safety [11], emotions [12,13,14,15], sleep quality [16], self-efficacy [15,17], and perceived health [18]. The effects of access to these resources are systematically smaller on incident chronic disease [12,14,16], all-cause mortality [13,17,19], and cause-specific mortality [20] for Blacks compared to Whites.

These differential effects are found for several resources and health outcomes [21,22,23,24]. That is, regardless of their type, these effects are systematically smaller for Blacks than Whites [25,26,27,28,29,30,31,32,33,34]. Diminished return for Blacks seems robust and hold independent of setting, cohort, age group, psychosocial determinants, and health outcome [35,36]. These findings are derived from national studies such as the Americans’ Changing Lives (ACL) study [18], the Midlife in the United States (MIDUS) study [14], the Religion, Aging, and Health Survey (RAHS) [19], the National Survey of American Life (NSAL) [23], and the National Health Measurement Study (NHMS) [21]. As all these longitudinal cohort studies have recruited a national sample, their results are generalizable to the United States population [1].

To give a few examples, high education credentials have failed to reduce the risk of physical inactivity [37], obesity [37], depressive symptoms [38], and suicidal ideation [39] among Blacks. Among Black men, high educational attainment is associated with an increase in depressive symptoms over time [38]. Black women with high educational attainment report higher suicidal ideation [39]. As education level increases, Blacks do not gain as much self-rated health as their White counterparts [2]. Another study found a weaker health effect of educational attainment for Blacks than for Whites [40]. All these studies support the Blacks’ “diminishing returns” hypothesis [1,2].

There are, however, certain social resources that may have stronger effects for Blacks than Whites. Blacks report higher levels and experience greater health benefits from each unit of religious involvement and spirituality [41,42]. Krause [43,44,45] and others [46] have documented stronger health effects of religious involvement for Blacks than Whites. Lincoln et al., has also shown that social support better protects mental health for Blacks than Whites [47]. Dupre et al. have shown how religious attendance may be one of the mechanisms explaining the Black-White mortality crossover [41]. Hummer and colleagues have shown that high levels of religious attendance increase life expectancy by 7 and 13 years for Whites and Blacks, respectively [48,49]. Keyes et al., have shown that despite all of their stress and adversity, Blacks have a higher chance of “flourishing” than Whites (i.e., high levels of psychological well-being and low rates of mental illness) [42,50]. Kessler has shown that any given stressor has more negative effects on the health of Whites than Blacks [51]. Assari has shown that religious social support fully mediates the effect of church attendance on mental health of Whites than Blacks [52]. 

Although systematic reviews have shown that having more social connections (both objective and subjective aspects) increases life expectancy [53], it is still unknown whether or not racial groups similarly gain life expectancy from having more social contacts. Research has shown that the health gain from social connectedness is comparable with the well-established health gain from physical activity, immunization, access to health care, and health promoting behaviors [53]. Social contacts increase access to a number of social, psychological, and behavioral assets that promote health and increase longevity [54,55,56,57,58,59,60]. Social contacts also provide tangible and emotional social support, which both protect health [61].

## 2. Aim

Despite the growing evidence on “Blacks diminished returns” and systemic differences in the effects of resources on health of Whites and Blacks [1,2], less is known about such phenomena for the health effects of social contacts [53]. The current study aimed to assess whether the life expectancy gain associated with social contacts over a long follow up differs for Blacks and Whites. To provide generalizable results, data from a national longitudinal study with a representative sample of American adults were used. 

## 3. Methods

### 3.1. Design and Setting

Data came from Americans’ Changing Lives (ACL), a 25-year longitudinal study from 1986 to 2011 [62]. The ACL is a nationally-representative U.S. study of more than three thousand American adults who were at least 25 years old [62]. More description on sampling and data collection is available elsewhere [63,64].

### 3.2. Ethics

The study procedures were in compliance with the Helsinki Declaration of 1975. All participants provided informed consent. The study protocol was approved by the University of Michigan Institutional Review Board (IRB).

### 3.3. Sampling and Participants

The study recruited a stratified multistage probability sample of adults. All participants were limited to noninstitutionalized American adults who were at least 25 years old and were living in the continental U.S. in 1986 (representing 70% of sampled households and 68% of sample individuals at baseline). The study oversampled older adults (age > 60) and Blacks. Although the study enrolled 3617 adults, current analysis is limited to 3361 individuals. From this number, 2205 were Whites and 1156 were Blacks.

### 3.4. Measures

*Demographic factors*. Demographic characteristics included age and gender. Age and gender were treated as continuous and categorical variables, respectively. For gender, male was the referent category. 

*Socioeconomic status*. Socio-economic characteristics included educational attainment, income, and employment, all collected in 1986. Education was defined as years of schooling treated as a continuous measure. Income was measured as an 11-category measure of the income of the respondent (and spouse if present). Income was treated as a continuous variable. Employment was a dichotomous measure [10].

*Race*. Participant’s race was measured at baseline (1986) using multiple survey items. Participants were asked, “In addition to being American, what do you think of as your ethnic background or origins?” and then were asked, “Are you White, Black, American Indian, Asian, or another race?” Participants who responded with more than one racial group were then asked to identify which one would best describe their race. ACL also assessed the state or foreign country of birth as well as Hispanic Ethnicity. Using all these items, the following race categories were built: (1) Non-Hispanic White; (2) Non-Hispanic Black; (3) Non-Hispanic Native American; (4) Non-Hispanic Asian; and (5) Hispanic. This study, however, only include the Non-Hispanic Whites and Non-Hispanic Blacks.

*Depressive Symptoms*. Frequency of depressive symptoms was measured using the Center for Epidemiological Studies-Depression scale (CES-D, 11 item) [65]. CESD measures the extent to which an individual feels depressed, happy, lonely, sad, and joy. Other items measure whether everything was an effort, their sleep was restless, people were unfriendly, they did not feel like eating, people dislike them, they could not get going. Item responses ranged from “never or hardly ever” (score 1) to “most of the time” (score 3). Positively worded items were reverse-coded. A mean score was calculated that was standardized and then treated as a continuous measure. Higher score was indicative of greater frequency of depressive symptoms. Abbreviated CES-D scales are shown to be reliable and valid [66,67,68].

Health Behaviors.

Obesity.

*Chronic Medical Conditions (CMC)*. Baseline number of CMC was measured using self-reported data. Participants were asked whether a health care provider has ever told them they have any of the following seven focal conditions: cancer, diabetes, hypertension, heart disease, stroke, chronic lung disease, and arthritis. A total score was calculated resulting in a score with a potential range from 0 to 7 [62,64].

*Social Contacts*. Social contact was measured in 1986 using a single item. The question was “How often do you get together with friends, neighbors or relatives and do things like go out together or visit in each other’s home?” Multiple studies such as Changing Lives of the Older Couples (CLOC) [69], National Health and Nutrition Examination Survey (HNES) [70], and the Tecumseh (Michigan) Community Health Study [70], have used the same item to measure social contact/social engagement. This measure is derived from the Berkman-Syme Social Network Index (SNI) [69] which measures frequency of contact with friends and family members. SNI scores predict all-cause mortality of men and women net of socioeconomic status, physical activity, obesity, smoking status, alcohol intake, health care use, and health status [69]. This item’s score ranges from 0 to 6, with 0 representing the highest and 6 reflecting the lowest levels of social engagement [70,71].

*Mortality*. Mortality data from 1986 through 2011 were obtained through the following three sources: National Death Index (NDI), death certificates, and informants. In most cases, death information was verified with death certificates. Mortality data were evaluated regardless of the follow up status of each participant. With a handful of exceptions, the death status of all participants could be determined. For the handful number of cases where death could not be verified with death certificates, all the information was reviewed carefully. Actual death was certain in all cases. Only in a few cases, date of death was ascertained from the informants or the NDI report. Overall, 1737 deceased Black or White participants were detected. The remaining 1624 Black or White individuals survived to the end of 2011.

## 4. Statistical Analysis

To accommodate the complex sample design of the ACL data, we used Stata version 12.0 for data analysis. Standard errors (SE) were estimated using Taylor series linearization. Thus, our calculated SEs reflect the survey design, and associated sampling and non-response baseline weights.

For multivariable analysis, we estimated a series of Cox proportional hazards models. These models require the following two variables as outcome: (1) an event; and (2) time to the event. Mortality was coded as 1 if all-cause death happened at any time between 1986 and 2011. Mortality was coded 0 otherwise. Time to death (or censoring) was calculated as the number of months from baseline to time of death, loss to follow up, or the end of the study (year 2011).

In the first step before running Cox proportional hazard models, we used -estat phtest- in Stata for Schoenfeld residual analysis to test the proportional hazards assumptions (non-significance of the overall model). 

The main predictor was social contacts at baseline (measured in 1986). Time interval from baseline to mortality was the outcome. Baseline demographic factors (age and gender), SES (education and income), health behaviors (smoking and drinking), and health status (CMC, obesity, and depressive symptoms) were covariates. Race was the focal moderator, defined as Blacks versus Whites (the referent category).

First, we ran models in the pooled sample to evaluate the additive and multiplicative effects of race and social contacts on mortality, while all covariates were controlled. To test if race and social contacts interact on mortality, we also ran a model with an interaction term. Finally, we ran our models stratified by race. Hazard ratios (HR) with 95% confidence intervals (CI) are reported. 

## 5. Results

Table 1 presents descriptive statistics of the pooled sample, as well as separately for Whites and Blacks. While Blacks and Whites did not differ in age and gender, Blacks had lower education, income, and employment compared to Whites. Blacks also had worse health status (CMC, obesity, and depressive symptoms) compared to Whites. Blacks reported less social contacts than Whites (Table 3).

Table 2 summarizes the results of a number of Cox proportional hazards models in the pooled sample. First model included only the main effects. The second model also included an interaction term between race and social contacts. The first model showed that social contacts predicted a higher life expectancy in the pooled sample. We also found a significant interaction between race and social contacts, indicating a stronger predictive role for Whites compared to Blacks (Table 2).

Table 3 reports the results of the proportional Cox models separately for Whites and Blacks. As this table shows, higher social contact was protective against risk of mortality among Whites but not Blacks (Table 3).

## 6. Discussion

Using a national sample of American adults, the current study aimed to compare Blacks and Whites for the effects of social contacts on life expectancy over a 25-year period. We found that the effect of social contacts and life expectancy is weaker for Blacks than Whites. Our finding that baseline social contacts better predict life expectancy for Whites than Blacks is in line with previous research [1]. 

The findings among Whites are nothing new [72]. Such findings are in line with the literature that suggests social connections (both objective and subjective aspects) increase life expectancy [53]. Social engagement increases access to a number of social, psychological, and behavioral assets that promote health and increase longevity [54,55,56,57,58,59,60]. 

More than a century ago, Durkheim showed that social engagement is protective for health [73]. From a sociological and psychological standpoint, the effects of social contacts can be explained by emotional and tangible social support. There are also several biological explanations behind the health effects of social contacts [74]. Positive and supportive social relationships are linked to lower cardiovascular reactivity [75,76], lower blood pressure [77,78], better regulation of inflammatory markers [79,80] and the stress system [76]. Individuals who receive adequate social support secrete lower levels of cortisol in response to stress as compared to the people receiving ineffective social support [76]. Social support may be beneficial because it decreases biological sensitivity to psychosocial stress, potentially shielding the individual from the harmful effects of stress-related increases in cortisol [76,81]. Thus, social support increases resilience to stress [81,82].

Review articles by Cohen and Uchino have shown that the evidence is robust on the effects of emotional social support on mental and physical health [61,83,84]. In addition to the effects of social support through reducing the impact of stress and enhanced mental health, social support also fosters a sense of meaning and purpose in life [85,86] and of personal control over life [87]. Social support also reduces risky behaviors [41,88]. All these mechanisms explain the positive effects of social contacts [89,90]. Social support also helps with management of chronic disease [91].

Mestrovic and Glassner coined the term Durkheimian hypothesis on stress [92], a sociological theory that explains how social engagement reduces the likelihood of illness. They argued that social integration underlies the stress-illness process. They used Durkheim’s work in the context of the homo duplex concept, which suggests social integration involves the interplay of individualism and social forces [92].

Social contacts also reduces health risk behaviors [88]. For example, Berkman and Breslow conducted a prospective study in Alameda County that showed that involvement with formal (e.g., religious organizations) and informal (e.g., friends and relatives) social ties is associated with more healthy behaviors over a 10-year period. Some of the protective effect of social ties on health behaviors may be due to peer effects, control, or group habits [93].

Not all effects of social contacts are, however, positive. Although social engagement provides enjoyment, stimulation, warmth, and support, social relations may also impose constraints and demands on the individual. So, both positive and negative aspects of social relations should be seen. Close relations may be associated with conflicts or social undermining. Social ties may also be very demanding. Some social gatherings may also involve unhealthy lifestyles, particularly for men [94,95,96].

The racial difference observed in this study is in line with the results of other studies from ACL [18], MIDUS [14], RAHS [19], NSAL [23], and NHMS [21]. All these studies have shown that resources and assets better promote health for Whites than Blacks [55]. These studies have shown similar patterns for the effects of education [9], employment [10], neighborhood safety [11], emotions [12,13,14,15] sleep quality [16], self-efficacy [15,17], and perceived health [18] on incident chronic disease [12,14,16], all-cause mortality [13,17,19], and cause-specific mortality [20], all being smaller for Blacks compared to Whites. These studies collectively suggest that there exists *Blacks’ diminished return* [2], which means minority status bounds the potential health gains from available psychosocial resources [3,4,5,6,7,8].

Previous research has shown that social support, religious involvement and spirituality may have a stronger effect on health of Blacks than Whites [43,44,45,48,49]. Different from the current findings, Assari [52], Lincoln [47], Krause [43], and others [46] have documented stronger health effects of religion involvement for Blacks than Whites. 

There is still a need to test whether psychosical resources have similar or group-specific effects on health. Williams, Kessler, Neighbors, and others have argued that researchers should systematically test potential interactions between race and resources on health [97,98]. Mehta has shown that behavioral and social risk factors do not have similar health effects across all social groups [99]. Kaufman, however, has discussed that due to the potential overlap between race and resources and due to residual confounding, it is challenging to decompose the effects of race and psychosocial resources on health [100].

Malat, Mayorga-Gallob, and Williams have suggested that there is a larger effect of social and psychological factors in Whites that may be due to their Whiteness (social privilege) [101]. Ceci and Papierno showed that resources are always more protective for the “Haves” than the “Have-Nots” [102].

If resources better serve Whites than Blacks, then several universal interventions—across different domains—may have the unintended effect of widening pre-existing gaps between disadvantaged and advantaged populations. That is a considerable risk if such interventions and programs are made available to all population groups, regardless of the fact that minorities may have more difficulty gaining benefits from such interventions [1,102]. Such universal interventions and policies have the potential of widening the gap. This is because socially and economically advantaged populations are readier than the disadvantaged group to gain health from resources [102,103]. Such interventions that have the potential to exacerbate the inequalities by disproportionately benefiting less disadvantaged groups are called Interventions-Generating Inequalities’ (IGIs) [104].

As equal resources result in unequal health gains for American Whites and American Blacks [1], social and public policies should not merely focus on equalizing the distribution of resources [1]—policies should also target the differential distribution of barriers across groups. Initial advantage (better access to economic resources and having a better health status and cognitive ability) leads to cumulative differences that means widening of pre-existing gaps over time [105]. For example, according to the cumulative advantage theory in the area of child development, initial advantage leads to further cumulative advantage and initial disadvantage being accentuated over time [80].

The current study had a number of limitations. First, a single item was used to measure social contacts in this study. The ACL measure of social contact was particularly limited as it conflates frequency of contacts with friends and associates with frequency of interaction with relatives (e.g., immediate and extended family members). Thus, the results should be tested for replication using more comprehensive measures of social engagement. Second, we did not conceptualize social contacts and other covariates (such as SES) as time varying covariates. This was because we were interested in the Black-White differences in the long-term effects of baseline social contacts, similar to other examples published before. Thus, we decided to focus on comparison of the slopes for baseline social contacts on 25-year mortality risk, without considering potential changes in SES or social contacts over time. Other researchers may expand or replicate our findings using other data sets. Finally, our study was limited to individual-level social engagement [96,106]. There is a need to study how race interacts with higher-level social cohesion [107] and residential segregation [106]. Despite these limitations, this was one of the first studies on racial differences on the long-term health gains from social contacts. Long term follow-up, large sample size, and nationally representative sample were strengths of this study.

## 7. Conclusions

In summary, social contacts increase life expectancy for Whites but not Blacks. This finding introduces social engagement as another social resource that differentially affects the life expectancy of Whites and Blacks.

## Figures and Tables

**Table 1:** Descriptive Statistics in the pooled sample and stratified by race.

	All		Whites		Blacks	
	Mean (SE)	95% CI	Mean (SE)	95% CI	Mean (SE)	95% CI
Age	47.77(0.534)	46.69–48.84	47.96(0.601)	46.75–49.17	46.33(0.717)	44.89–47.78
Education *	12.53(0.096)	12.34–12.73	12.69(0.105)	12.48–12.90	11.37(0.233)	10.90–11.84
Income *	5.41(0.093)	5.22–5.60	5.57(0.101)	5.36–5.77	4.25(0.183)	3.88–4.62
Depressive symptoms *	−0.03(0.025)	−0.08–0.02	−0.07(0.025)	−0.13–0.02	0.28(0.051)	0.18–0.38
Chronic Medical Conditions *	0.79(0.028)	0.74–0.85	0.78(0.031)	0.71–0.84	0.91(0.052)	0.81–1.02
Social Contacts (low)	3.46(0.036)	3.38–3.53	3.51(0.039)	3.44–3.60	3.00(0.059)	2.88–3.12
	% (SE)	95% CI	% (SE)	95% CI	% (SE)	95% CI
Gender						
Men	47.26(0.012)	44.86–49.68	47.82(0.013)	45.12–50.52	43.18(0.022)	38.79–47.69
Women	52.74(0.012)	50.32–55.14	52.18(0.013)	49.48–54.88	56.82(0.022)	52.31–61.21

^*^ *p* < 0.05 for comparisons between Blacks and Whites.

**Table 2:** Association between baseline social engagement and all-cause mortality overall.

	HR (SE)	95% CI for HR	HR (SE)	95% CI for HR
	Model 1		Model 2	
Race (Black)	1.14 #	1.00–1.30	1.38 *	1.06–1.79
Gender (female)	0.53 ***	0.46–0.62	0.54 ***	0.46–0.62
Age	1.08 ***	1.08–1.09	1.08 ***	1.08–1.09
Education	1.18 **	1.04–1.34	1.18 **	1.04–1.34
Income	1.25 **	1.07–1.46	1.25 **	1.08–1.46
Employment	0.69 **	0.56–0.84	0.69 ***	0.56–0.84
Smoking	1.92 ***	1.65–2.24	1.92 ***	1.65–2.24
Drinking	0.99	0.87–1.12	0.99	0.88–1.13
Obese	1.07	0.91–1.26	1.07	0.91–1.26
Depressive Symptoms	1.06	0.93–1.23	1.07	0.93–1.23
Chronic Medical Conditions	1.19 ***	1.13–1.25	1.19 ***	1.13–1.24
Social Contacts (low)	1.04 *	1.00–1.08	1.05 *	1.01–1.09
Social Contacts (low) × Race	-	-	0.93 *	0.87–1.00

Results are based on Cox proportional hazards models # *p* < 0.1, * *p* < 0.05, ** *p* < 0.01, *** *p* < 0.001.

**Table 3:** Association between baseline social engagement and all-cause mortality stratified by race.

	HR (SE)	95% CI for HR	HR (SE)	95% CI for HR
	Whites		Blacks	
Gender (Female)	0.51 ***	0.43–0.60	0.69 **	0.54–0.89
Age	1.09 ***	1.08–1.10	1.07 ***	1.05–1.08
Education	1.20 *	1.04–1.38	1.11	0.86–1.43
Income	1.23 *	1.04–1.46	1.34 *	1.01–1.77
Employment	0.68 **	0.54–0.86	0.73 #	0.53–1.01
Smoking	2.01 ***	1.67–2.42	1.53 ***	1.27–1.84
Drinking	1.00	0.87–1.16	0.99	0.75–1.32
Obese	1.05	0.86–1.29	1.14	0.91–1.44
Depressive Symptoms	1.09	0.93–1.28	0.92	0.74–1.15
Chronic Medical Conditions	1.21 ***	1.14–1.27	1.11 *	1.02–1.21
Social Contacts (low)	1.05 *	1.01–1.10	0.98	0.93–1.04

Results are based on Cox proportional hazards models # *p* < 0.1, * *p* < 0.05, ** *p* < 0.01, *** *p* < 0.001.
